# Potential protective role of GLP-1 receptor agonists for lithium-induced nephrotoxicity: a population-based observational study

**DOI:** 10.1017/S1092852925100709

**Published:** 2025-11-02

**Authors:** Roger S. McIntyre, Angela T.H. Kwan

**Affiliations:** 1 Department of Psychiatry, University of Toronto, Toronto, ON, Canada; 2 Department of Pharmacology and Toxicology, University of Toronto, ON, Canada; 3 Faculty of Medicine, University of Ottawa, Ottawa, ON, Canada

**Keywords:** Lithium, semaglutide, glucagon-like peptide-1 receptor agonists (GLP-1RA), lithium-induced nephrotoxicity, renal adverse events, bipolar disorder, pharmacovigilance, depression, kidney, mood stabilizer

## Abstract

**Objectives:**

We hypothesized that semaglutide, a glucagon-like peptide-1 receptor agonist (GLP-1 RA) and treatment currently U.S. Food and Drug Administration (FDA) approved to reduce worsening of kidney disease and kidney failure may protect against lithium-induced nephrotoxicity.

**Methods:**

Renal adverse events (AEs) reported to the FDA Adverse Event Reporting System (FAERS) between December 2003 and December 2024 were analyzed using the validated OpenVigil 2.1 platform. Reporting odds ratios (RORs) were computed for the MedDRA terms renal impairment, renal failure, chronic kidney disease (CKD), end-stage renal disease (ESRD), and acute kidney injury (AKI) in association with lithium, semaglutide, and their co-reporting. A disproportionality signal was considered statistically significant when *p* < 0.05, and the lower bound of the information component (IC_025_) was greater than 0.

**Results:**

Lithium was associated with significantly elevated reporting odds across all renal AEs, with each outcome exceeding statistical signal thresholds (*p* < 0.0001, IC_025_ > 0). In contrast, semaglutide demonstrated inverse associations for renal impairment, renal failure, CKD, and ESRD (RORs = 0.25–0.54), and a neutral association for AKI (ROR = 0.96); however, none met the criteria for a disproportionality signal (IC_025_ < 0). Co-reported use of lithium and semaglutide did not yield a significant signal for any renal outcome, including AKI (ROR = 5.81, *p* = 0.015, IC_025_ < 0).

**Conclusions:**

This observation, provides impetus to conduct adequate, thorough, mechanistic, clinical, and observational studies to determine whether semaglutide (and/or other incretin receptor agonists) could possibly mitigate the risk for, and/or modify the trajectory of, lithium-induced nephrotoxicity.

## Introduction

Lithium remains a first-line treatment for acute mania and for preventing relapse in bipolar disorder.^1^ ^,^^2^ However, long-term use carries a significant risk of kidney injury, with lithium-induced nephrotoxicity estimated to affect up to 20% of patients receiving chronic therapy.^3^ ^–^^5^ This complication is often progressive with continued lithium exposure and a not infrequent reason for treatment discontinuation. Pathology results typically show chronic tubulointerstitial nephropathy with evidence of tubular atrophy, interstitial nephritis and fibrosis as well as cortical microcysts.^6^ ^,^^7^ The mechanisms implicated are hypothesized to include inflammatory and oxidative stress pathways.^8^ Although treatments exist for lithium-induced nephrogenic diabetes insipidus, no therapy has yet been established to meaningfully prevent or slow the disease trajectory of lithium-induced nephrotoxicity.

Glucagon-like peptide-1 receptor agonists (GLP-1 RAs) are FDA approved in the treatment of multiple medical conditions including, but not limited to, type 2 diabetes mellitus and obesity with acute- and long-term effects that have exceeded traditional agents. In addition to their metabolic effects, GLP-1 RAs have received U.S. Food and Drug Administration (FDA) approval for metabolic-dysfunction-associated steatohepatitis, obstructive sleep apnea, and reducing the progression of chronic kidney disease (CKD), end-stage kidney disease, and cardiovascular mortality in adults with type 2 diabetes and CKD.^9^ Mechanisms implicated in the renal protective effects of GLP-1 RAs include suppression of activated inflammatory effectors; decreased renal immune cell infiltration; regulation of innate immune activity; decreased receptor expression for advanced glycation end product (RAGE); inhibition of nuclear factor κB (NF-κB) signaling; and reductions in fibrosis, oxidative stress, and autophagy. Beneficial effects in microvasculature function and neural signaling are also implicated.^10^

The nephroprotective mechanisms of GLP-1 RAs introduce a testable and viable hypothesis that we preliminarily address in this study, ie, whether semaglutide, a GLP-1 RA currently approved by the FDA for reducing the risk of worsening kidney disease and kidney failure in adults with type 2 diabetes, may also exert protective and/or illness-modifying effects against lithium-induced nephrotoxicity. The aforementioned hypothesis derives from overlapping molecular mechanisms implicated in diabetic nephropathy and lithium-induced nephrotoxicity. A separate and related hypothesis is whether protective effects may be direct cytoprotective actions on molecular and cellular renal systems and/or indirectly by improvements in microvascular and metabolic function.^10^

## Methods

### Data source and extraction

Adverse event (AE) data were systematically retrieved from the U.S. FDA Adverse Event Reporting System (FAERS), a global post-marketing surveillance database that aggregates voluntary reports from healthcare professionals, manufacturers, and consumers. Reports submitted between December 2003 and December 2024 were analyzed using OpenVigil 2.1, a validated pharmacovigilance platform that interfaces with the openFDA API to provide structured access to FAERS.^11^ ^–^^20^ This system enables standardized data retrieval, filtering, deduplication, and preprocessing of large-scale safety datasets in alignment with international pharmacovigilance guidelines.

### Data cleaning and curation

Drug names were standardized according to the United States Adopted Name (USAN) convention, with cross-references to DrugBank and Drugs@FDA to resolve brand and generic discrepancies as well as abbreviations. Automated mapping and error-correction algorithms were used to harmonize terminology across entries. Reports that were incomplete, structurally invalid, or internally inconsistent were excluded. Potential duplicate cases were identified through matching of demographic and case-level variables and verified against the FDA DEMO table before removal.

### AE coding and case selection

All AEs were standardized using Preferred Terms (PTs) from the Medical Dictionary for Regulatory Activities (MedDRA) to ensure consistent classification of renal outcomes.^21^ Primary endpoints included the MedDRA terms renal impairment, renal failure, CKD, end-stage renal disease (ESRD), and acute kidney injury (AKI), selected for their documented association with lithium-induced nephrotoxicity. The final dataset underwent clinical review by experts from the FDA’s Center for Drug Evaluation and Research (CDER) and Center for Biologics Evaluation and Research (CBER) to confirm completeness, clinical plausibility, and coding accuracy.^22^ ^,^^23^

Analyses were limited to reports identifying lithium or semaglutide as the primary suspect drug to ensure accurate attribution of AEs to the drug of interest. In contrast, co-reported cases involving both lithium and semaglutide were queried without suspect filters to maximize sensitivity for detecting potential interaction signals, regardless of reporter designation.

### Signal detection and statistical analysis

Disproportionality analysis was performed using the reporting odds ratio (ROR), defined as the odds of a renal AE event occurring with a given drug relative to all other drugs in the FAERS database. To strengthen signal detection accuracy, a multitiered analytic framework was applied, consistent with validated pharmacovigilance methodologies from prior studies.^13^ ^,^^17^ ^24^ ^,^^25^Specifically, the Evans criteria (*n* > 2, χ^2^ > 4, ROR > 2) were used alongside calculation of the lower bound of the 95% credibility interval (IC_025_).^26^ A signal was considered statistically significant when IC_025_ > 0 and *p* < 0.05. In addition to ROR, proportional reporting ratio (PRR) and relative risk ratio (RRR) were calculated as secondary validation metrics.

To minimize reporting biases, we combined frequentist and Bayesian disproportionality methods with standardized signal thresholds to enhance the accuracy and stability of signal detection. The Bayesian IC_025_ metric helps reduce the impact of notoriety bias—where increased awareness temporarily boosts reporting—and the Weber effect, which reflects early post-marketing spikes in AE submissions. This stabilization occurs through Bayesian shrinkage, which adjusts observed-to-expected ratios toward the overall reporting mean, reducing the influence of random variation, small sample sizes, and short-term reporting fluctuations.^27^ ^,^^28^ Visualization of RORs, including forest plots, was performed using RStudio (version 2023.06.1 + 524, “Desert Sunflower” release).

### Ethical considerations

Institutional review board (IRB) approval was not required because FAERS is a publicly accessible de-identified database.

## Results

**Figure 1.**
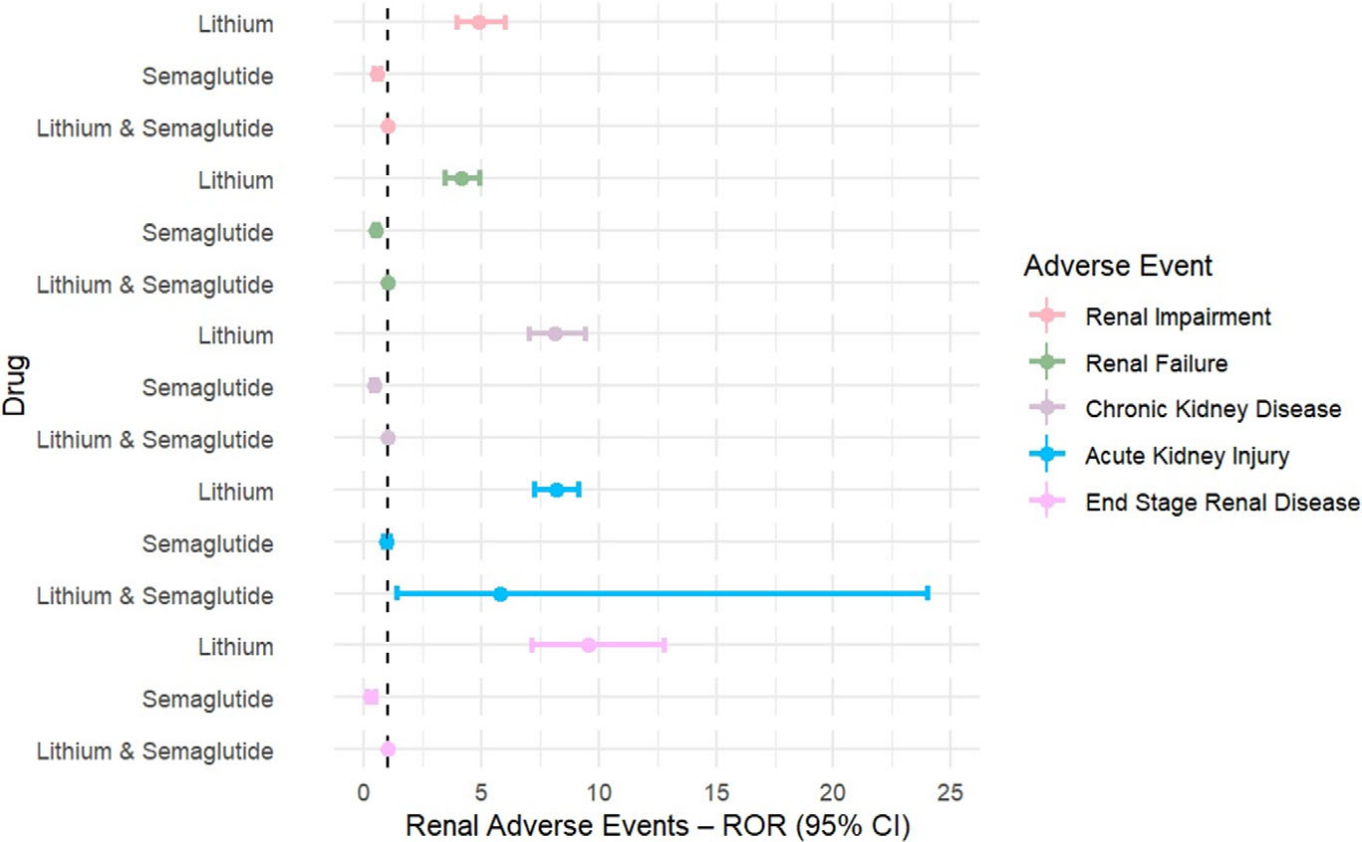
Forest plots of RORs were generated for lithium, semaglutide, and their combination across key renal adverse events.

Lithium was consistently associated with significantly elevated reporting across all renal AEs. Disproportionality estimates were robust and exceeded signal thresholds for every endpoint: renal impairment (ROR = 4.98, 95%CI = 4.03–6.15, *p* < 0.0001, IC_025_ = 1.91), renal failure (ROR = 4.11, 95%CI = 3.43–4.92, *p* < 0.0001, IC_025_ = 1.69), CKD (ROR = 8.15, 95%CI = 7.02–9.45, *p* < 0.0001, IC_025_ = 2.70), ESRD (ROR = 9.55, 95%CI = 7.14–12.77, *p* < 0.0001, IC_025_ = 2.62), and AKI (ROR = 8.17, 95%CI = 7.28–9.17, *p* < 0.0001, IC_025_ = 2.74), as summarized in Table 1 and illustrated in Figure 1.

**Table 1.** Renal Adverse Events Associated with Lithium, Semaglutide, and Their Combination Compared to All Other Drugs in FAERS

	Target drug		Control								
	AE	All other AEs	AE	All other AEs	Rate (DE/D)	RRR (95% CI)	PRR (95% CI)	ROR (95% CI)	χ2	Evan criteria*	*p*-Value (IC025)
Renal impairment											
Lithium	88	4767	48,910	13,184,046	1.81%	4.90 (3.98–6.03)	4.90 (3.99–6.03)	4.98 (4.03–6.15)	270.12	Y	<0.0001 (1.91)
Semaglutide	64	31,697	48,934	13,157,116	0.20%	0.54 (0.43–0.70)	0.54 (0.43–0.70)	0.54 (0.43–0.69)	24.09	N	<0.0001 (−1.29)
Lithium and Semaglutide	0	43	48,998	13,188,770	0.0%	–	–	–	0.73	N	–
Renal failure											
Lithium	122	4733	82,539	13,150,417	2.51%	4.02 (3.38–4.80)	4.03 (3.38–4.80)	4.11 (3.43–4.92)	276.09	Y	<0.0001 (1.69)
Semaglutide	101	31,660	82,560	13,123,490	0.32%	0.51 (0.42–0.62)	0.51 (0.42–0.62)	0.51 (0.42–0.62)	47.68	N	<0.0001 (−1.30)
Lithium and semaglutide	0	43	48,998	13,188,770	0.0%	–	–	–	0.73	N	–
Chronic kidney disease											
Lithium	181	4674	62,614	13,170,342	3.73%	7.86 (6.81–9.07)	7.88 (6.83–9.09)	8.15 (7.02–9.45)	1082.23	Y	<0.0001 (2.70)
Semaglutide	63	31,698	62,732	13,143,318	0.20%	0.42 (0.33–0.54)	0.42 (0.33–0.53)	0.42 (0.33–0.53)	50.79	N	<0.0001 (−1.67)
Lithium and semaglutide	0	43	62,795	13,174,973	0.0%	–	–	–	0.43	N	–
Acute kidney injury											
Lithium	311	4544	109,946	13,123,010	6.41%	7.69 (6.91–8.57)	7.71 (6.92–8.59)	8.17 (7.28–9.17)	1819.46	Y	<0.0001 (2.74)
Semaglutide	254	31,507	110,003	13,096,047	0.80%	0.96 (0.85–1.09)	0.96 (0.85–1.09)	0.96 (0.85–1.09)	0.39	N	0.51 (−0.27)
Lithium and semaglutide	2	41	110,255	13,127,513	4.65%	5.58 (1.44–21.61)	5.58 (1.44–21.61)	5.81 (1.41–24.01)	3.67	N	0.015 (−1.05)
End-stage renal disease											
Lithium	46	4809	13,245	13,219,711	0.95%	9.44 (7.08–12.59)	9.47 (7.10–12.63)	9.55 (7.14–12.77)	339.05	Y	<0.0001 (2.62)
Semaglutide	8	31,753	13,283	13,192,767	0.025%	0.25 (0.13–0.50)	0.25 (0.13–0.50)	0.25 (0.13–0.50)	17.21	N	0.0001 (−3.14)
Lithium and semaglutide	0	43	13,291	13,224,477	0.0%	–	–	–	4.84	N	–

In contrast, semaglutide showed inverse associations for renal impairment (ROR = 0.54, 95%CI = 0.43–0.69, *p* < 0.0001, IC_025_ = −1.29), renal failure (ROR = 0.51, 95%CI = 0.42–0.62, *p* < 0.0001, IC_025_ = −1.30), CKD (ROR = 0.42, 95%CI = 0.33–0.53, *p* < 0.0001, IC_025_ = −1.67), and ESRD (ROR = 0.25, 95%CI = 0.13–0.50, *p* = 0.0001, IC_025_ = −3.14), while the association with AKI was neutral (ROR = 0.96, 95%CI = 0.85–1.09, *p* = 0.51, IC_025_ = −0.27). None of the semaglutide outcomes met the disproportionality signal threshold (IC_025_ < 0).

Co-reporting of lithium and semaglutide produced no estimable RORs for renal impairment, renal failure, CKD, or ESRD because of zero reported cases. For AKI, 2 co-reported cases were identified (ROR = 5.81, 95%CI = 1.41–24.01, *p* = 0.015, IC_025_ = −1.05); however, this also did not meet the statistical signal threshold (IC_025_ < 0).

## Discussion

In this large population-based pharmacovigilance study, and in accordance with lithium’s known nephrotoxic effects, lithium use was associated with significantly elevated reporting odds of renal impairment, acute renal failure, CKD, and ESRD. We also observed that concomitant administration of semaglutide with lithium did not yield a corresponding increase in nephrotoxicity signals. This observation potentially provides preliminary support for the hypothesis that GLP-1 RAs may exert a potentially protective effect on lithium-associated renal injury. Over the past 2 decades, longitudinal studies and registry analyses have revised prior estimates of lithium-induced nephropathy, indicating that while the absolute risk may be lower than historically reported, lithium exposure remains a cause and contributor to CKD progression and a not infrequent reason for treatment discontinuation.^3^ ^,^^29^ ^–^^31^

GLP-1 RAs are increasingly prescribed for metabolic conditions that differentially affect individuals living with bipolar disorder, including obesity, type 2 diabetes mellitus, obstructive sleep apnea, and CKD.^32^ ^–^^34^ Beyond their metabolic benefits, GLP-1 RAs have demonstrated reductions in cardiovascular morbidity and mortality, which is the leading contributor to excess and premature mortality in bipolar disorder.^35^ Moreover, GLP-1 RAs have been investigated for use in psychotropic-drug-related weight gain, binge eating, metabolic liver disease, and substance- or alcohol-use disorders, all of which are clinical presentations that are frequently encountered in the bipolar population.^36^ ^–^^40^

The molecular and cellular dynamics affected by GLP-1 RAs have separately introduced a hypothesis that these agents may hold therapeutic potential in the treatment and prevention of mental disorders and progressive cognitive impairment.^41^ For example, GLP-1 RAs are known to promote neurogenesis, neuroplasticity, and autophagy while reducing inflammation, oxidative stress, and neuronal apoptosis within critical neural circuits.^41^ ^–^^43^ The molecular, cellular, and circuit-level effects attributed to GLP1-RAs may confer neuroprotective effects against the progression of illness, particularly with respect to cognitive functioning.^44^

However, our findings must be carefully and cautiously interpreted, as they are regarded as highly exploratory and hypothesis-generating. The overarching reason for a measured interpretation of our findings is that analyzing data in a pharmacovigilance dataset (eg, FAERS) is not capable of establishing causality, and such databases are not intended to inform deliberation as to whether protective effects exist. Disproportionality analyses, including ROR and IC_025_, quantify reporting patterns relative to background rates and do not measure clinical risk or incidence.^45^ Accordingly, an IC_025_ value below zero indicates an absence of disproportionality rather than a signal of protection.^45^ These metrics are meant to detect safety signals, not infer benefit; an inverse IC_025_ reflects a reporting trend rather than therapeutic protection and requires validation in patient-level studies.^46^ These findings, therefore, cannot be interpreted as evidence that GLP-1 RAs confer a true renoprotective effect.

The limitations of our analysis, as well as any inference, interpretation, or implementation messaging derive from our methodology. Herein, we used the US FAERS, which relies on spontaneous reporting from the general population as well as industry sponsors (for whom reporting is mandatory).^47^ Spontaneous reporting of any AE cannot be considered a comprehensive estimate of its occurrence. Moreover, FAERS does not include information related to variables that would be required before cause and effect can be established (ie, the Bradford Hill criteria).^48^ In addition, we are not able to know, in the general population, what percentage of persons prescribed lithium have also been prescribed a GLP-1 RA, including semaglutide. Notwithstanding this limitation, the prescription of GLP-1 RAs has increased approximately 700% in the US during the past 4 y, and approximately 12% of US adults have been prescribed GLP-1 RAs, with 6% of the population prescribed semaglutide.^49^ ^,^^50^ In addition, as stated earlier, GLP-1 RAs are indicated and/or prescribed off-label for many conditions known to differentially affect persons living with depressive and bipolar disorders.^36^ Consequently, it can be assumed that a percentage (albeit unknown percentage) of persons prescribed lithium may also have been co-prescribed a GLP-1 RA.

In addition to the aforementioned points, multiple other limitations should be acknowledged. First, the absence of denominator data precludes estimation of event incidence or risk ratios, as the total number of exposed individuals (to lithium, GLP-1 RAs, or both) is unknown. Second, confounding by indication is likely, as GLP-1 RAs are prescribed primarily to individuals with type 2 diabetes or obesity, who differ from lithium-treated patients in metabolic risk, renal surveillance intensity, and access to nephrology care. Third, confounding by indication may introduce channeling bias, since patients with advanced renal disease are often excluded from GLP-1 RA therapy, potentially creating an artificial appearance of protection. Fourth, FAERS is inherently susceptible to multiple reporting biases—including notoriety bias, where regulatory or media attention increases reporting; the Weber effect, characterized by heightened reporting shortly after drug approval; and surveillance bias due to differential clinical monitoring. Fifth, co-reporting cases were extremely limited, yielding low statistical power and wide uncertainty intervals that preclude meaningful inference.

Furthermore, the broader limitations of spontaneous reporting systems must be recognized. FAERS data depend on voluntary submissions from clinicians, patients, and manufacturers, resulting in underreporting and variable data completeness. The database also lacks patient-level covariates such as baseline renal function, diabetes severity, medication dose, duration of exposure, and comorbid conditions—all of which are essential for causal inference. Temporal trends in drug utilization and awareness may further influence reporting frequency, adding uncertainty to observed associations. Consequently, these results should be regarded strictly within the descriptive and hypothesis-generating scope of pharmacovigilance rather than as confirmatory evidence.

The hypothesis that semaglutide and/or other GLP-1 RAs are protective against lithium-induced nephrotoxicity cannot be considered proven by our results. Instead, our results provide preliminary support for the viable and testable hypothesis of renal protection, and it is our hope that they provide impetus for comprehensive mechanistic, histopathologic, and clinical studies with adequate methodology. It also needs to be clearly stated that GLP-1 RAs have multiple AEs and safety concerns, which reduce acceptability and have been difficult to access for eligible persons due to acquisition cost barriers. In addition, early reports of suicidality associated with these agents resulted in regulatory investigations concluding that cause and effect cannot be established but should continue to be a variable for further evaluation.^24^ ^,^^51^ ^–^^53^

In conclusion, our overarching aim is to encourage comprehensive assessments of whether semaglutide (and/or other incretin receptor agonists) is capable of exerting nephroprotective effects in persons exposed to lithium. Definitive evidence would require triangulation of histopathologic, clinical, and observational studies with adequate methodology to determine cause and effect. Analyses based on pharmacovigilance data are inadequate to address causality and can neither confirm nor refute potential benefits of incretin receptor agonists in this context. Nonetheless, the absence of significant co-reporting of lithium nephrotoxicity and semaglutide—though potentially unrelated—could be aligned with an as yetunconfirmed hypothesis that incretin receptor agonists may be renally protective against lithium-associated injury.

## Data Availability

All data relevant to the study are included in the article.
